# The fetal pain paradox

**DOI:** 10.3389/fpain.2023.1128530

**Published:** 2023-03-21

**Authors:** Bridget Thill

**Affiliations:** University of Mary, Bismarck, ND, United States

**Keywords:** fetal pain, fetal analgesia, fetal anesthesia, fetal nociception, fetal awareness, subplate

## Abstract

Controversy exists as to when conscious pain perception in the fetus may begin. According to the hypothesis of cortical necessity, thalamocortical connections, which do not form until after 24–28 weeks gestation, are necessary for conscious pain perception. However, anesthesiologists and neonatologists treat age-matched neonates as both conscious and pain-capable due to observable and measurable behavioral, hormonal, and physiologic indicators of pain. In preterm infants, these multimodal indicators of pain are uncontroversial, and their presence, despite occurring prior to functional thalamocortical connections, has guided the use of analgesics in neonatology and fetal surgery for decades. However, some medical groups state that below 24 weeks gestation, there is no pain capacity. Thus, a paradox exists in the disparate acknowledgment of pain capability in overlapping patient populations. Brain networks vary by age. During the first and second trimesters, the cortical subplate, a unique structure that is present only during fetal and early neonatal development, forms the first cortical network. In the third trimester, the cortical plate assumes this function. According to the subplate modulation hypothesis, a network of connections to the subplate and subcortical structures is sufficient to facilitate conscious pain perception in the fetus and the preterm neonate prior to 24 weeks gestation. Therefore, similar to other fetal and neonatal systems that have a transitional phase (i.e., circulatory system), there is now strong evidence for transitional developmental phases of fetal and neonatal pain circuitry.

## Introduction

Controversy exists as to when conscious pain perception in the fetus may begin. Currently, two hypotheses prevail that are distinguished by a demarcating line at 24 weeks gestation. First, according to the hypothesis of cortical necessity, functional thalamocortical projections to the somatosensory cortex that develop after 24–28 weeks gestation are required before conscious pain perception is possible (1, 2). Second, the subplate modulation hypothesis holds that functional activity in the cortical subplate and/or subcortical structures is sufficient to mediate pain perception in the fetus before 24 weeks gestation (3), and possibly as early as 12 weeks gestation (4). The subplate, a transient layer located beneath the cortical plate in the developing cortex, forms the predominant cortical circuitry from the first through third trimesters (5).

Determining the onset of pain perception is important, as invasive procedures affect both the fetus and the preterm neonate before 24 weeks, prompting consideration of analgesia (pain relief) and anesthesia (loss of physical sensation with or without loss of consciousness). In clinical practice, observable and measurable behavioral, hormonal, and physiologic indicators of pain, prior to 24 weeks gestation, have guided the use of analgesics in neonatology, fetal surgery, and fetal anesthesiology for decades. Pain is acknowledged and treated in the earliest preterm neonates <24–28 weeks using validated pain assessment tools (6). Fetal surgeons administer direct fetal analgesia and anesthesia as early as 15–16 weeks gestation (7, 8). Fetal anesthesiologists recommend the use of fetal anesthesia from the second trimester onward (>14 weeks gestation) (9), or in all invasive maternal-fetal procedures regardless of gestational age, in order to “inhibit the humoral stress response, decrease fetal movement, and blunt any perception of pain [(10), p. 1167].”

**Figure 1:**
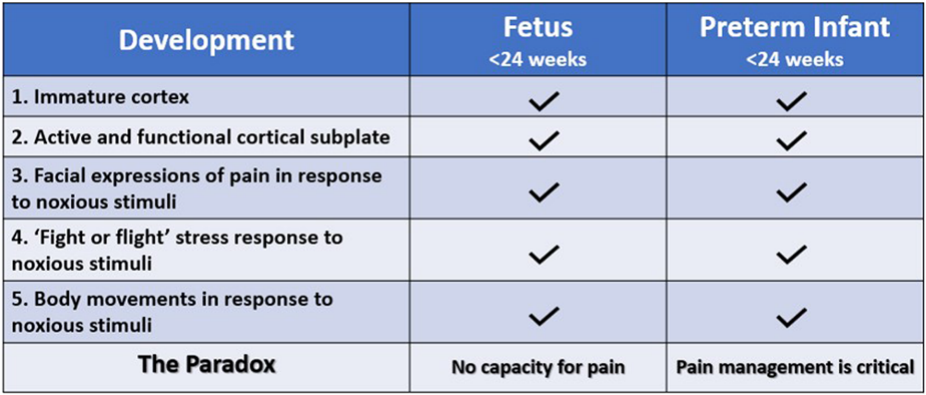
The fetal pain paradox. Both the fetus and preterm infant <24 weeks gestation have an immature cerebral cortex (12) and an active, functional cortical sublate (13, 14). Both mount hormonal and hemodynamic stress responses (6, 15) and demonstrate pain-related facial expressions (6, 16) and body movements (6, 15) following noxious stimuli. The standard of care for preterm infants, according to the American Academy of Pediatrics, is pain management utilizing validated pain assessment tools. However, the American College of Obstetricians and Gynecologists (11), the Society for Maternal Fetal Medicine (1), and the Royal College of Obstetricians and Gynaecologists (2) state that pain perception is not possible until after 24–28 weeks gestation.

Conversely, the American College of Obstetricians and Gynecologists (ACOG), the Society for Maternal Fetal Medicine (SMFM), and the Royal College of Obstetricians and Gynaecologists (RCOG) state that (1) pain perception requires a comprehensive network of neural connections to the cerebral cortex that is not possible until at least 24–25 weeks and unlikely until after 28 weeks gestation; (2) accepted behavioral and physiologic indicators of pain in the extremely preterm infant (<28 weeks) are reflexive or spontaneous and not indicative of a pain experience; and (3) the use of anesthesia or analgesia in neonatal and prenatal surgery serves purposes unrelated to pain, such as preventing long-term consequences of stress responses and decreasing fetal movement (1, 2, 11). Thus, a paradox exists in the disparate acknowledgment and treatment of pain perception by different medical groups in neonates <24–28 weeks gestation and fetuses of similar age (Figure 1).

Comparison between the preterm infant and the age-matched fetus is supported by similarities between these two populations. The fetus and the preterm infant share a predominantly fetal physiology with an immature cortex and an active cortical subplate. Both exhibit pain-related responses to noxious stimuli including body movements, facial expressions, and hormonal and physiologic responses (Figure 1). Both may also exhibit “freeze and dive” behavioral and physiologic responses to painful stimuli, in which the fetus and preterm infant become immobilized, and observable responses to noxious stimuli are muted or absent due to lack of energy reserves or underlying physiologic stress (17–20). Differences between the extrauterine and intrauterine environments also affect such comparisons. First, very low gestational age infants are generally in a critical state of health requiring intensive care, while the age-matched fetus *in utero* is generally in a state of homeostasis. Second, different sensory experiences in the extrauterine environment, particularly medically-indicated noxious procedures (ie. heel lances), may affect neurodevelopment. Studies show that premature infants with at least 40 days in the neonatal intensive care unit (NICU) have increased neuronal responses to noxious stimuli compared to healthy neonates born at the same corrected age (21). While comparison between preterm neonates and fetuses has limitations, much can be learned from their shared anatomy and physiology, as compared to adult or animal studies.

## Differences in early development

Nociceptive pathways in the fetus and preterm infant differ from an older infant and adult in several ways, including (1) the presence of the subplate in the first through third trimesters (22); (2) the lack of descending inhibitory pathways (Descending Pain Modulatory System, DPMS) to mitigate pain until post term development, resulting in a fetus that is “extremely sensitive to painful stimuli [(23), p. 1031, (24)];” (3) large receptive fields, resulting in low pain threshold and poor pain localization (25, 26), such that noxious stimuli to the foot, for example, may be perceived as affecting the entire leg; and (4) increased vulnerability of the developing nervous system to painful procedures experienced early in life, resulting in an increased risk of harmful long-term sequelae such as altered pain sensitivity and neurocognitive development, including impairment in cognition, learning disorders, attentional disorders, behavioral problems, and motor abnormalities (6, 10, 20, 27, 28).

There is noted caution in attributing conscious pain perception to nonverbal populations. Pain is a subjective experience that, without verbal report, can only be inferred from behavioral, physiological, and neural markers (29). However, it is generally acknowledged in terminology and clinical practice, that *pain* in extremely preterm infants is underassessed and undertreated, with an urgent need to improve *pain* assessment and management in the youngest premature infants (19, 27, 29–31). A paradox exists not only in the disparate acknowledgment of pain perception by different medical and scientific groups, but also in the disparate use of pain terminology attributed to the nonverbal preterm infant, but not to the age-matched fetus, in whom references to nociception predominate.

## An evolving understanding of pain

**Figure 2:**
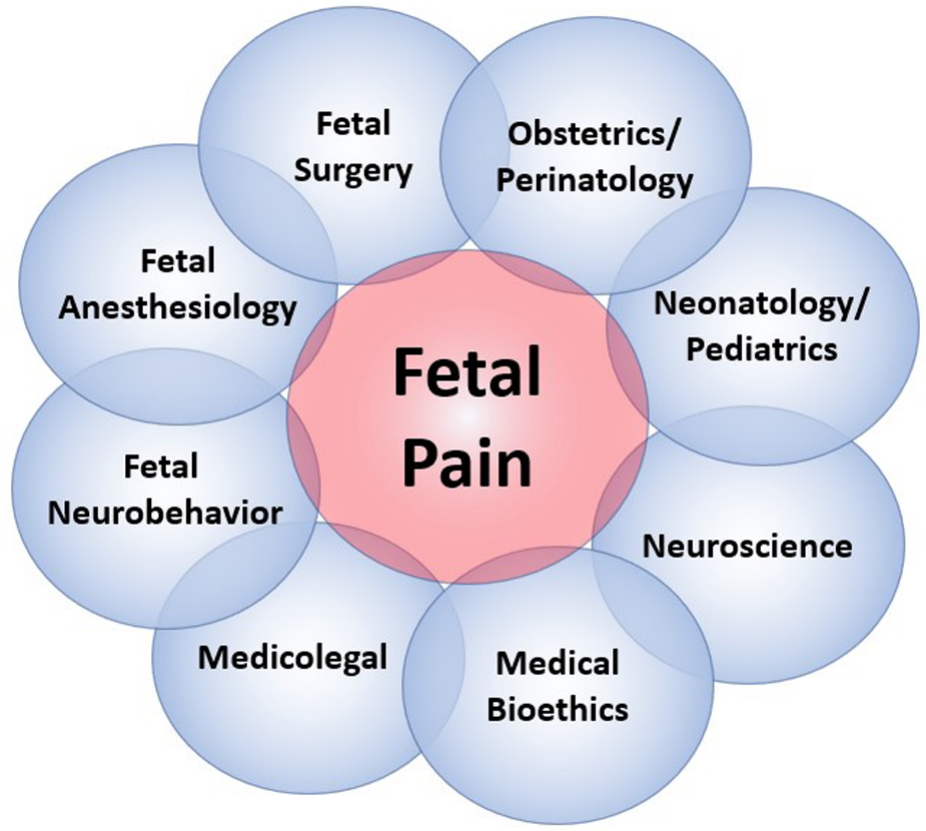
The multidisciplinary dimensions of fetal pain research.

**Figure 3:**
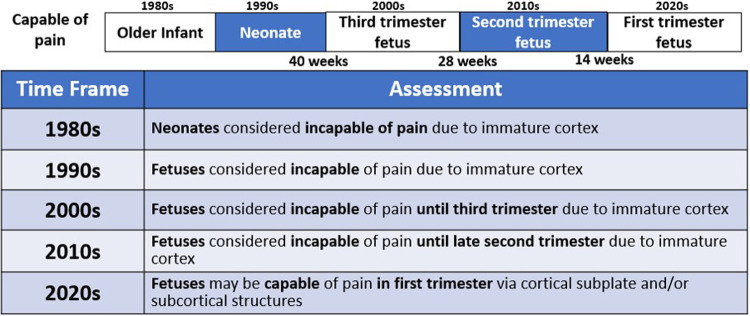
An evolving understanding of pain. Scientific understanding and recognition of pain capacity in the neonate and fetus have evolved over time. In the 1980s and 1990s, medical consensus held that neonates (32, 38) and fetuses (15) lacked the capacity to perceive pain. In the 200s (12), 2010s (34), and 2020s (4, 35, 36), the understanding of fetal pain capacity has shifted.

The field of fetal pain research is complex and multidisciplinary, with unique perspectives offered from a variety of domains (Figure 2). Scientific understanding and evidence-based medicine change over time, particularly regarding the ability of the infant and the fetus to experience pain (Figure 3).

Until the 1980s, early studies of neurologic development concluded that neonates and young infants lacked the brain structures and connections necessary for pain perception (32, 37, 38). Surgery on infants was frequently performed with paralysis, but without pain management, with catastrophic outcomes (33). During bedside invasive procedures, clinicians suspended consideration of observable pain indicators in view of neurological studies that had concluded that neonatal pain was impossible (37, 38).

In the 1990s, fetuses of all gestations were considered incapable of pain and invasive fetal procedures were conducted without analgesia or anesthesia, until studies demonstrated fetal cardiovascular and hormonal stress responses to invasive procedures (15, 39–41). The field of fetal anesthesiology arguably began in 2001 after research demonstrated that fetal responses to noxious stimuli were attenuated by analgesics (42, 43). Researchers demonstrated that fetal stress responses (cortisol and β-endorphin) do not occur with needling of the non-innervated umbilical cord. However, a significant increase in these stress hormones does occur with needling through the innervated fetal trunk accompanied by vigorous body and breathing movements(15). These physiologic and behavioral responses are nullified by the use of analgesics (42), resulting in a fetus that is “still and appears quiescent and calm [(4), p. 6].” These fetal studies mirrored neonatal studies from the 1980s which (1) showed that analgesics blunt hormonal stress responses to surgery (44); and (2) began the era of neonatal pain management (33, 38).

**Figure 4:**
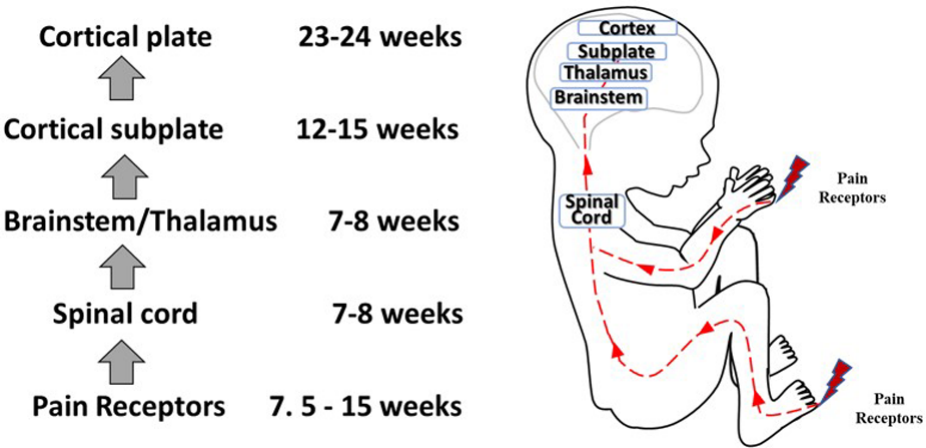
Development of nociceptive pathways. Peripheral pain receptors develop in most areas of the fetus between 7.5–15 weeks gestation (48). Afferents reach the spinal cord (49), the brainstem, and thalamus by 7–8 weeks (50, 51). Thalamic projections to the cortical subplate emerge at 12–15 weeks (14, 34, 52) and to the cortical plate after 23–24 weeks gestation (53).

In the 2000s, studies concluded that fetal pain did not develop until the third trimester (>28 weeks) due to a lack of cortical function (12). In the 2010s, researchers determined that the physiologic capacity to perceive pain developed during the second trimester (14–28 weeks), ranging from before (23, 45) and after thalamocortical connectivity at 24 weeks (34). In the 2020s, researchers suggest that the necessity of the cortex in pain perception may have been overestimated (4, 16, 35, 46). Recent evidence indicates that thalamic projections to the subplate at 12 weeks gestation may be functionally equivalent to thalamocortical connections that develop at 24 weeks gestation (4), signifying that fetal pain mediated by the subplate and subcortical structures may be possible as early as the first trimester (<14 weeks) (Figure 4) (4, 35, 36, 47). Fetal responses to noxious stimuli during clinically-indicated procedures are listed in Table 1. Notably, published prospective studies of fetal responses to noxious stimuli have only been conducted in Europe (15, 39, 40, 42, 55) and South America (16, 57, 58), despite the exponential increase in fetal surgeries, particularly in North America.

**Table 1:** Fetal responses to noxious stimuli.

Source	# studied & GA	Noxious stimulus^a^	Fetal Response
Giannakoulopoulos et al. (1994) (15)	*N* = 16 23–29 weeks GA	Needling of IHV *via* puncture of fetal trunk	– Significant hormonal stress response to invasive needling: median increase in *β*-endorphin 590% and cortisol 183% – Vigorous body and breathing movements
Petrikovsky and Kaplan (1995) (54)	*N* = 7 15–18 weeks GAc(Case series)	Inadvertent contact of amniocentesis needle with fetal limb	– Brisk withdrawal of the involved part (except in one fetus with limb paralysis)
Teixeira et al. (1996) (39)	*N* = 28 18–36 weeks GA (Pilot study)	Needling of IHV *via* puncture of fetal trunk	– Significant decrease in MCA PI in response to transgression of fetal trunk, consistent with redistribution of blood supply to the brain (brain-sparing effect)
Giannakoulopoulos et al. (1999) (41)	*N* = 42 18–37 weeks GA	Needling of IHV *via* puncture of fetal trunk	– Significant elevation in fetal noradrenaline with needling involving transgression of fetal trunk – Dislodgement of needle in two cases of IHV needling due to vigorous fetal movements
Teixeira et al. (1999) (40)	*N* = 130 (136 procedures) 15–37 weeks GA	Needling procedures involving transgression of fetal trunk^b^	– Significant decrease in MCA PI within 70 s after painful stimulation, consistent with redistribution of blood supply to the brain (brain-sparing effect)
Fisk et al. (2001) (42)	*N* = 16 20–35 weeks GA	IHV transfusion *via* transgression of fetal trunk, with or without fentanyl	– Direct fetal analgesia blunts the hormonal and hemodynamic stress response to intrahepatic vein needling (β-endorphin and MCA PI responses, respectively)
Gitau et al. (2001) (55)	*N* = 51 18–35 weeks GA	Fetal blood sampling and intrauterine transfusion at IHV *via* piercing of fetal trunk; compared to maternal blood samples	– Fetal stress response to IHV transfusion, but not to transfusion at PCI (non-innervated); – Fetal responses are independent of maternal responses; – Fetal β-endorphin and cortisol responses are apparent from 18 to 20 weeks gestation, respectively
Mayorga-Buiza et al. (2017) (56)	*N* = 1 24 weeks GA (Case study)	Open fetal surgery for myelomeningocele repair, inadvertently initiated without administration of fetal anesthesia	– Fetal bradycardia; – Fetal recovery after epinephrine and administration of direct fetal anesthesia
Bernardes et al. (2018) (57)	*N* = 1 32 weeks GA (Case report)	Preoperative anesthetic injection into fetal thigh	– 10 facial actions coded by blinded investigators, before and after anesthetic puncture – pre-puncture score: 0–1/10; post-puncture score, 8–10/10
Bernardes et al. (2021) (58)	*N* = 13 28–33 weeks GA	Preoperative anesthetic injection into fetal thigh	– Fetuses demonstrate discriminative facial expressions in response to painful stimuli – Presence of five out of seven pain-related facial expressions discriminated pain from nonpainful startle and rest
Bernardes et al. (2022) (16)	*N* = 1 23 weeks GA	Preoperative intramuscular anesthetic injection into fetal thigh	– Facial expressions of acute pain demonstrated following intramuscular injection – Rated 5 out of 7 on fetal pain score by blinded investigators

GA, gestational age; IHV, intrahepatic vein; PCI, placental cord insertion; MCA PI, middle cerebral artery pulsatility index; wk, weeks.

^a^ Fetuses were exposed to noxious stimuli during clinically-indicated procedures.

^b^ Needling procedures involving transgression of fetal trunk: shunt insertion, tissue biopsy, ovarian cyst aspiration, urine aspiration, drainage of ascites, and fetal blood sampling and intrauterine transfusion *via* intrahepatic vein.

## Pain and nociception: definitions and development

The definition of pain established by the International Association for the Study of Pain (IASP) in 1979 states that pain is an “unpleasant sensory and emotional experience associated with actual or potential tissue damage, or described in terms of such damage (59).” In 2020, revisions to the IASP definition described pain as an “unpleasant sensory and emotional experience associated with, or resembling that associated with, actual or potential tissue damage,” noting that “verbal description is only one of several behaviors to express pain; inability to communicate does not negate the possibility that a human or a nonhuman animal experiences pain [(60), p. 1977].”

As noted by the IASP, in the preverbal population, several behaviors express pain and its unpleasantness. In the extremely preterm neonate (<28 weeks gestation), validated pain assessment tools utilize behavioral indicators of pain, including pain-related facial expressions, body and limb movements, and changes in breathing patterns, as well as physiologic indicators of pain (6). These indicators are likewise present in the fetus in response to noxious stimuli and occur in both the fetus and extremely preterm infant prior to thalamocortical connectivity (Figure 1). The use of age-dependent multimodal pain assessment tools, rather than univariate analysis, increases the sensitivity and specificity of detecting pain in preverbal populations, avoids underestimation of pain, and allows discrimination between responses to noxious and innocuous stimuli (19, 20, 30, 31, 61).

The IASP defines nociception as “the neural process of encoding noxious stimuli,” while the consequences of nociception may include autonomic and behavioral responses, as well as pain perception (62). Nociception, the neural transmission of noxious signals, does not always result in pain sensation, as in cases of general anesthesia or spinal cord transection in which transmission of signals to the brain is blocked or prevented (12, 63). Anesthesiologists note that pain perception typically occurs concomitantly with stress responses, such that pain is unlikely if hormonal stress responses are absent (64). In the fetus, hormonal stress responses are absent to minimal when analgesia is used during invasive procedures, leading researchers to consider the possibility of pain perception in the fetus once noxious-evoked stress responses are evident (Table 1) (42, 55, 65, 66).

**Figure 5:**
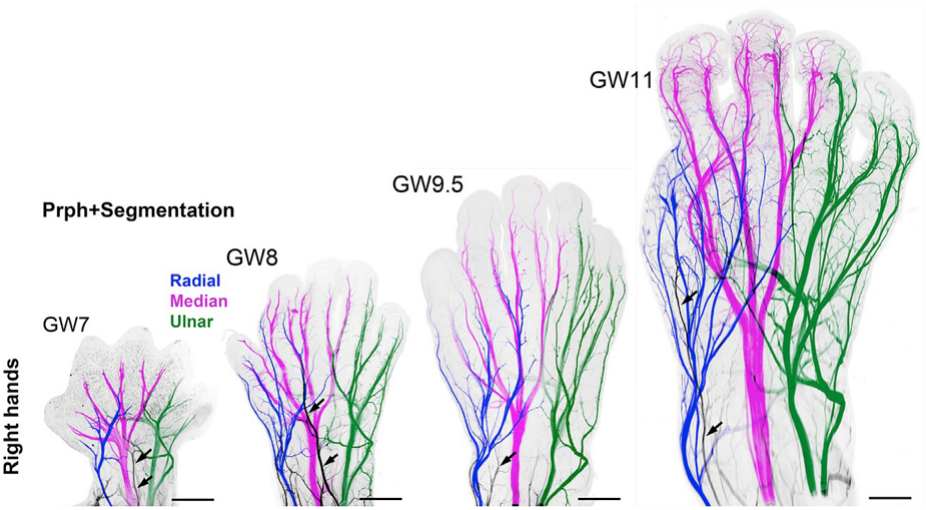
3D analysis of the sensory innervation of the developing human hand (67). Time series illustrating the developing innervation of sensory nerves of the right hand from GW7–GW11, labeled for the neuron-specific intermediate filament protein peripherin (Prph). Individual segmentation of the radial (blue), median (magenta), and ulnar (green) nerves are shown. The musculocutaneous nerve (arrows) transiently extends into the hand. Prph, neuron-specific intermediate filament protein peripherin; 3D, three-dimensional; GW, gestational week.

Questions remain as to (1) when nociception in the developing fetus triggers a conscious perception of pain; and (2) whether the cortex after 24 weeks or the subplate/subcortical structures prior to 24 weeks are sufficient for the pain experience. Nociceptive pathways, extending from peripheral receptors to the brain emerge during early fetal development, reaching the brainstem, thalamus, and cortical subplate by 12 weeks gestation (Figure 4) and the cortical plate after 24 weeks gestation. Notably, the thalamus relays all afferent sensorimotor information (excluding olfaction) first to the subplate and later to the cortical plate. Peripheral sensory receptors develop in most areas of the fetus between 7.5–14 weeks gestation (48). Figure 5 demonstrates the developing sensory innervation in the human hand from 7 to 11 weeks gestation (67). Peripheral afferents reach the spinal cord (49), brainstem, and thalamus by 7–8 weeks gestation (50, 51). The first thalamic projections to the subplate arrive at 12–15 weeks gestation (34, 52, 68), earlier than the 20–22 weeks cited in older studies (69, 70). After 23–24 weeks gestation, thalamocortical fibers project to the cortical plate, particularly to layer 4 (L4) of the developing somatosensory cortex (12, 53).

**Figure 6:**
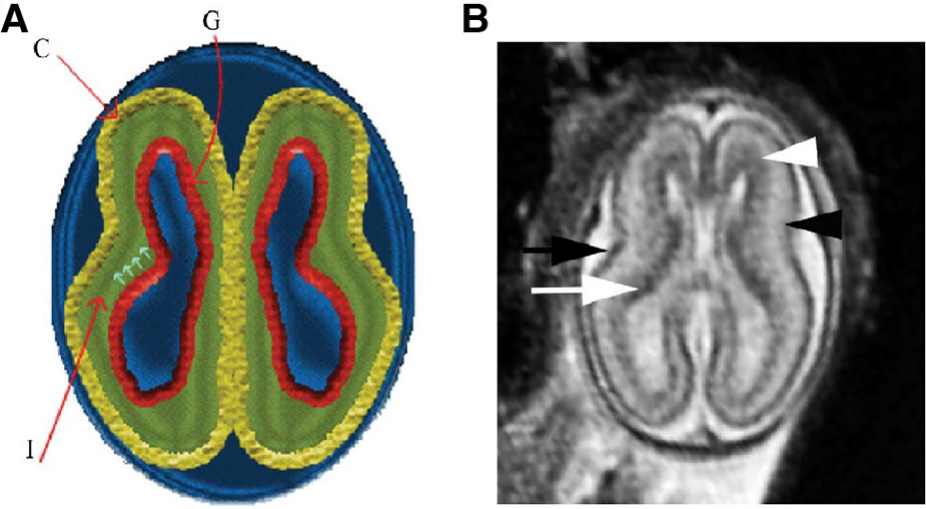
Normal multilayered magnetic resonance imaging (MRI) appearance of fetal brain early in gestation (72). (**A**) A diagram representing the fetal brain at 19 weeks of gestation shows smooth surface and multilayered appearance of the parenchyma with an inner germinal matrix (G), intermediate layer (I), and a developing cortex (**C**). The small arrows point to the direction of the migrating neurons from germinal matrix to the developing cortex. (**B**) Axial balanced fast field echo MR image of a normal brain at 19 weeks of gestation shows a smooth surface and multilayered parenchyma with an inner hypointense germinal matrix (white arrow), an intermediate layer, and an outer hypointense developing cortex (black arrow). Two additional sublayers can be identified: subventricular zone (white arrowhead) and subplate (black arrowhead). Subventricular zone is thick in the frontal region and shows slightly hypointense signal as it contains germinal matrix with increased cell production. The subplate zone appears slightly hyperintense as it has high water content because of extracellular matrix.

The subplate, discovered in 1974, is a transient layer located beneath the cortical plate in the developing cerebral cortex, expanding to four times the thickness of the cortical plate by mid-gestation (71). MRI of the fetal subplate at 19 weeks gestation is shown in Figure 6 (black arrowhead) (72). Neurons from the subplate then migrate to their mature position in the cortex, and the subplate largely disintegrates between 3 months preterm and 3 months post-term (22), while the cortical plate forms layers 2–6 of the developing cortex (73).

Two behavioral responses to noxious stimuli are discussed in more detail below: pain-related facial expressions and limb movements/withdrawal in response to noxious stimulation.

## Noxious-Evoked facial expressions and limb movements

Facial expressions are a cornerstone of neonatal pain assessment and are recognized as sensitive and specific predictors of the presence and severity of pain, despite the brainstem origins of these markers (74), particularly when multiple facial movements are assessed. Per the American Academy of Pediatrics, only five neonatal pain scales have been rigorously tested in extremely preterm infants <24–28 weeks gestation; all utilize facial expressions to assess pain (6).

Facial expression-based pain scales, such as the Neonatal Facial Coding System, score 9 distinct facial movements in order to discriminate pain from non-pain states in extremely preterm infants (<28 weeks). Other validated neonatal pain scales use fewer facial expressions but in combination with physiologic, behavioral, and contextual indices. At early gestational ages, multimodal pain assessment tools are critical in differentiating signs and symptoms of pain from those attributable to other causes (6). For example, the Premature Infant Pain Profile-Revised (PIPP-R), a composite behavioral and physiologic pain scale for preterm neonates from 25 weeks gestation, consists of 7 items: 3 measures of facial expressions of pain (brow bulge, nasolabial furrow, and eye squeeze), 2 physiological indices (heart rate and oxygen saturation) and 2 contextual parameters (gestational age and behavioral state), scored at three levels. The range of scores indicates minimal to no pain (<7), moderate pain (7–12), or severe pain (>12) (75). Ranges with cut-off values such as these have proven effective in discriminating pain from non-pain states at early gestational ages (76, 77).

In 2019, Green and colleagues utilized a partial PIPP-R pain scale, (using 3 measures of facial expressions, but not physiologic or contextual parameters) in scoring neonatal responses to a noxious heel lance vs. a control heel lance which did not pierce the skin. The study concluded that facial expressions did not reliably distinguish noxious from non-noxious stimuli in the earliest gestational ages studied (28 weeks) compared to late preterm infants (>33 weeks) (28). A subsequent study in 2022 disputed these findings, noting that the use of multidimensional pain assessment tools (facial expression, brain activity, heart rate, and limb withdrawal) discriminated noxious from non-noxious procedures with an accuracy of 78%–79% in 28–31 week preterm infants. This underscores the need for a multimodal approach to acute pain assessment, including physiologic, behavioral, and contextual parameters, particularly at earlier gestational ages (6).

**Figure 7:**
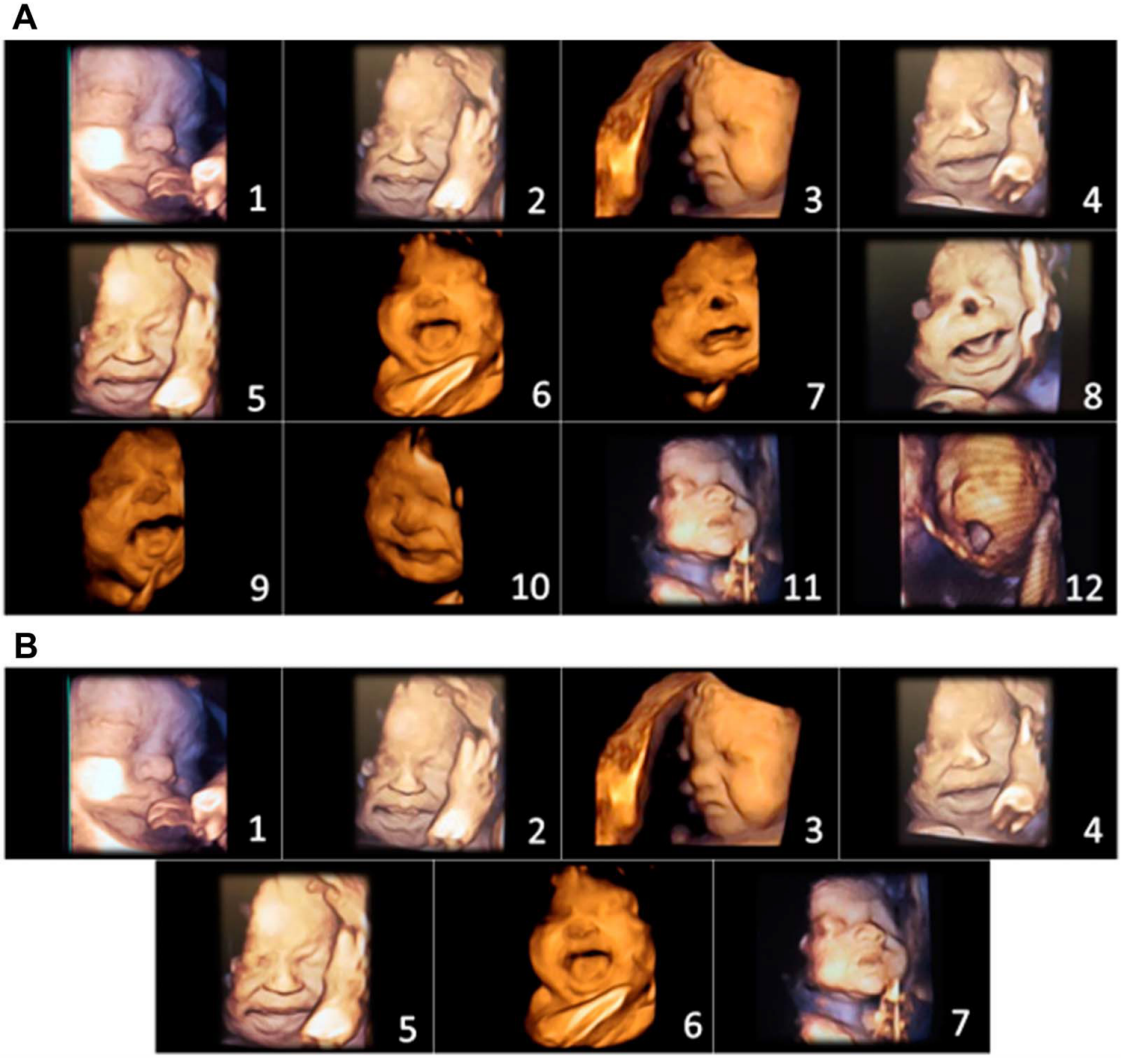
Pain assessment tool for third trimester fetuses during anesthetic injection into the thigh during fetal surgery (58). (**A**) Initial items from neonatal facial coding system and 2 supplementary items. 1. Brow lowering. 2. Eyes squeezed shut. 3. Deepening of the nasolabial furrow. 4. Open lips. 5. Horizontal mouth stretch. 6. Vertical mouth stretch. 7. Lip purse. 8. Taut tongue. 9. Tongue protrusion. 10. Chin quiver. 11. Neck deflection 12. Yawning. (**B**) Final items from the Fetal-5 Scale. 1. Brow lowering. 2. Eyes squeezed shut. 3. Deepening of the nasolabial furrow. 4. Open lips. 5. Horizontal mouth stretch. 6. Vertical mouth stretch. 7. Neck deflection.

**Figure 8:**
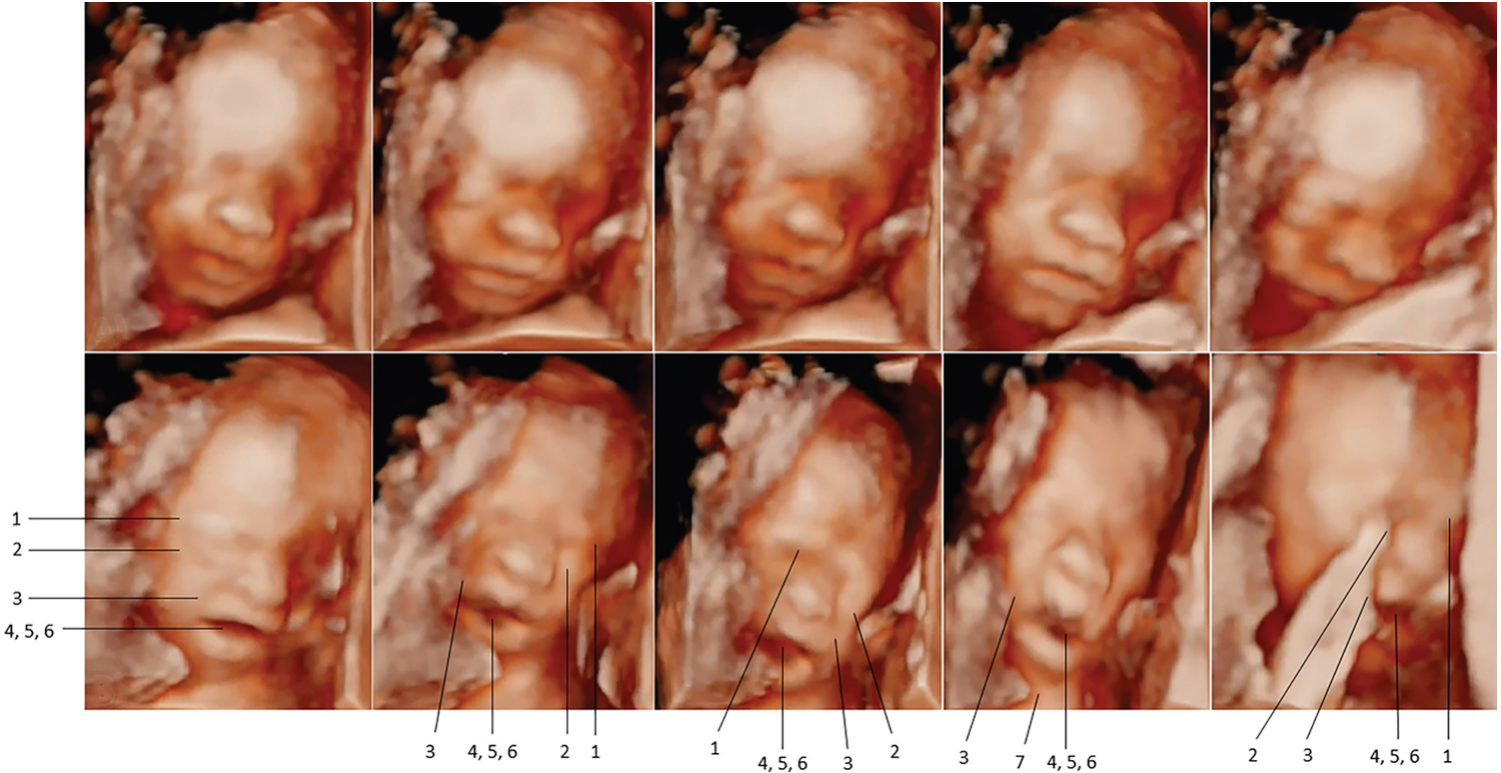
Pain-related facial expressions in 23-week fetus in response to intramuscular injection of fetal thigh, analyzed by blinded investigators (16). Four-dimenstional ultrasound images of fetal facial expressions analyzed before (upper row) and after (lower row) anesthetic puncture, demonstrating the lack of pain-related facial response before and the presence of pain-related facial expressions after the painful stimulus. Seven criteria were considered to be indicative of fetal pain response: 1, brow lowering; 2, eyes tightly shut; 3, deepening of the nasolabial furrow; 4, open lips; 5, vertical mouth stretch; 6, horizontal mouth stretch; and 7, neck extension.

In 2021 and 2022, Bernardes and colleagues utilized another pediatric pain scale, the Neonatal Facial Coding System, to score 7 different fetal facial expressions in response to anesthetic puncture of the fetal thigh during intrauterine surgery. Blinded investigators analyzed 4D-US images before and after the anesthetic puncture. These researchers concluded that both second and third trimester fetuses respond to noxious stimuli with pain-related facial expressions and that a cutoff value of 5 out of 7 facial expressions effectively discriminated pain from nonpainful auditory stimuli (16, 58). Figures 7, 8 show noxious-evoked facial expressions at 31 weeks and 23 weeks, respectively. Figure 7 highlights the need for modified pain scales at earlier gestational ages. Paradoxically, the Society for Maternal Fetal Medicine and the Royal College of Obstetricians and Gynaecologists state that these fetal facial expressions are reflexes and “do not reflect any experience of pain or suffering (1,B4; 2),” even though they are accepted indicators of pain in age-matched neonates (6).

Several neonatal pain scales use limb movements in response to noxious stimuli as a pain indicator. Fetal responses to noxious stimuli also include withdrawal of the limbs (Table 1).

Reflex withdrawal is often dismissed by proponents of cortical necessity as a spinal cord-mediated response, not indicative of supraspinal processing. Recent research regarding reflex limb withdrawal notes that in term infants (with cortical plate connectivity) withdrawal from noxious stimuli strongly correlates with nociceptive brain activity in the cortical plate, indicating that noxious limb withdrawal occurs concomitantly with transmission of pain signals to the brain (78). Further evidence of correlation in preterm infants (who have subplate, but not cortical plate, connectivity) is needed. No known studies have similarly investigated the relationship between noxious-evoked reflex withdrawal in preterm infants and activation of the subplate and subcortical circuitry. Additionally, some researchers discount reflex limb withdrawal as a nondiscriminative pain marker due to lack of differentiation between tactile and painful stimulation in preterm infants (79). However, research by Gursul and colleagues in 2019 found that the magnitude of limb withdrawal is discriminative, with a greater response occurring with noxious stimulation compared to tactile stimulation, as measured by EMG (20). Likewise, more intense noxious stimuli, such as intramuscular injections which are quantified as causing severe pain, trigger higher behavioral reactivity scores and discriminative facial expressions compared to noxious procedures of lower pain intensity such as heel lances (80).

## Fetal responses to noxious stimuli

Fetal pain research began in the 1990s, shortly after recognition of neonatal pain, in fetuses as early as 16 weeks gestation. Fetal therapeutic interventions, generally involving puncture of the fetal trunk, provided the occasion to evaluate noxious-evoked responses in the fetus during invasive procedures (Table 1). Fetal responses to noxious stimuli include sizeable biochemical and circulatory hormonal and hemodynamic stress responses by 16–20 weeks gestation, which are blunted by analgesics (42). These responses include significant increases in stress hormones (β-endorphin 590% and cortisol 183%), vigorous body and breathing movements (15), significant elevation of noradrenaline (41), and significant decreases in middle cerebral artery pulsatility index consistent with a brain-sparing response (39, 40). Administration of direct opioid fetal analgesia prevented these responses (42). Fetal stress responses occur independently of maternal responses (55). Two studies documented responses to inadvertent noxious stimulation: (1) contact of the amniocentesis needle with fetal limbs, leading to brisk limb withdrawal (54); and (2) intrauterine surgery inadvertently initiated without fetal anesthesia resulting in bradycardia, which resolved after administration of epinephrine and fetal anesthesia (56). The fetus may mount a similar hormonal stress response under conditions of hypoxemia, such as when the umbilical cord or placenta is strangulated or ablated. During hypoxemic or noxious events, the fetus also may exhibit a freeze and dive response, characterized by inhibition of movements, bradycardia, and redistribution of blood flow to vital organs (18).

Primary studies of fetal responses to noxious stimulation utilizing 4D-US are novel. In 2018, an experimental model to assess and quantify acute pain responses during intrauterine surgery was described for the first time (57). As previously mentioned, Bernardes and colleagues utilized a modified neonatal pain assessment scale, the Neonatal Facial Coding System, to analyze 7 pain-related facial expressions in the fetus during anesthetic puncture of the fetal thigh. Blinded investigators rated fetal facial expressions pre and post-injection in fetuses from 23 weeks gestation. The study concluded that fetuses demonstrate discriminative facial expressions of acute pain following intramuscular injection (Figures 7, 8) (16, 58). Researchers emphasized the need for continuous monitoring of fetal activity and responses to invasive fetal procedures to better assess and treat procedural and post-procedural pain (3).

Though utilization of analgesia and anesthesia during fetal surgery began in the early 1980s and an anesthesiology and fetal therapy consensus statement in 2021 recommends administration of fetal anesthesia in all invasive maternal-fetal procedures (10), optimal anesthetic techniques and dosages continue to evolve. In 2022, a systematic review of anesthesia for fetal operative procedures concluded that several anesthesia approaches are utilized with no standardized protocols or dosage regimens based on the type of fetal procedure. A lack of standardized intraoperative fetal monitoring was also noted (81).

Anesthetic management during invasive fetal procedures employs a range of modalities with varying degrees of placental transfer and safety profiles (10). The use of maternal general anesthesia, particularly during open fetal surgery, allows for the transfer of anesthetics to the placental circulation, however, the need for higher doses of volatile anesthetic agents can have a substantial adverse impact on fetal hemodynamics and prolonged use raises concern of fetal neurotoxicity. Supplemental maternal intravenous anesthesia to reduce the dosage of volatile agents used during general anesthesia may lower this risk and allow adequate transplacental transfer (82). However, maternal anesthesia *via* local anesthetic infiltration or neuraxial blockade (ie. epidural anesthesia) may be preferred during fetal interventions. Though some transplacental transfer may occur, direct fetal anesthesia *via* intramuscular or intravenous administration is recommended in order to reliably blunt the fetal stress response to invasive procedures and to facilitate fetal pain relief and appropriate fetal positioning (10, 83, 84). Direct fetal anesthesia generally includes a cocktail of an opioid analgesic (i.e.. fentanyl), a nondepolarizing muscle relaxant (i.e., rocuronium) to achieve fetal immobility, and an anticholinergic agent (i.e., atropine) to minimize the risk of fetal bradycardia and is recommended for all surgeries on innervated tissue (85).

## Noxious-evoked brain activity

The use of neural markers in the preterm neonate has the potential to link functional neuroimaging to clinically observable pain-related measures to increase the sensitivity and specificity of pain assessment tools and to help discriminate pain from non-pain states. Modalities such as electroencephalography (EEG), functional magnetic resonance imaging (fMRI), magnetoencephalography (MEG), and near-infrared spectroscopy (NIRS) have been studied to determine brain activity at rest and during noxious and non-noxious stimulation in early neurologic development (19, 20, 86–88).

Neurological pain signatures (NPS) of noxious-evoked brain activity hold promise for the future, though numerous technical, ethical, and design issues currently preclude the diagnostic utility of such neural measures of pain (1, 89, 90). The use of noxious-specific brain activity, as a surrogate measure of pain, is particularly challenging for several reasons: (1) brain networks vary by age, with a subplate-dominant network in the first to third trimesters and a cortical-dominant network post-term (22), leading to differing patterns of neural activity by developmental stage; (2) inferring pain perception based on functional activity or inactivity within brain regions is difficult (27); (3) pain is a dynamic process, with neural responses affected by a variety of factors, including physiologic stress and gestational age (20, 89). Researchers conclude it is highly unlikely that a neural marker will capture the dynamic nature of pain in its entirety (19, 27, 89). Instead, clinicians and neuroscientists recognize the necessity of composite, multimodal pain assessment tools to increase predictive value, potentially including developmentally-specific neural measures, in preventing the underdiagnosis and undertreatment of pain (29) and in discriminating between pain and non-pain states (20, 31, 61, 91).

## The emergence of consciousness

Pain perception depends not only on transmission of nociceptive signals to the brain but also on the level of consciousness of the fetus or neonate. Definitions of consciousness, however, are diverse and elusive. Various definitions require (1) extrauterine life (92); (2) the presence of a longer memory span associated with second-order learning (88); (3) thalamocortical connections confirmed *via* electrophysiologic studies (93); or (4) body awareness after 25 weeks gestation (94). Notwithstanding, consciousness may be defined more fundamentally as the state of wakefulness and awareness (95).

Animal research by Mellor and colleagues in the 2000s suggested that the fetus is not awake until after birth due to the sedative effects of endocrine neuroinhibitors *in utero* (18, 34, 92). Recent research, however, has discredited this hypothesis, noting that (1) human endocrine neuroinhibitors do not confer any anesthetic effect in the human fetus, but only with artificial injection at high dosages; and (2) the fetus is arousable and responsive to external stimuli (96). Fetal studies have also demonstrated arousability and responsiveness (Table 1 and Figures 7, 8), leading to questions about the onset of fetal awareness.

**Figure 9:**
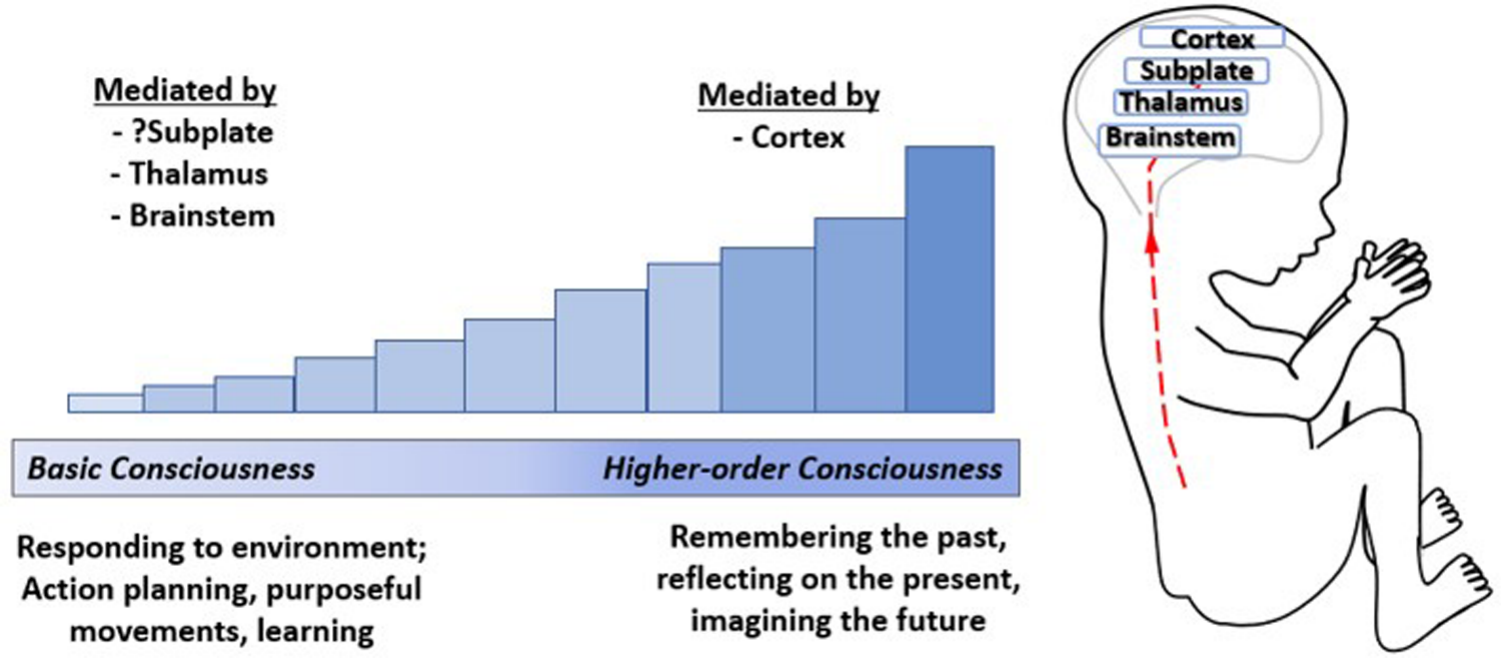
The emergence of consciousness. Consciousness has been likened to a dimmer switch beginning with a minimum basic level of consciousness mediated by the brainstem (100), thalamus (99), and possibly the cortical subplate (4) increasing to higher-order consciousness mediated by the cortex (100). Basic consciousness requires responsiveness to the environment (101), demonstrated by action planning, purposeful movements, and leaming (100), while higher-order consciousness involves memory, self-reflection, and imagining the future (98).

Conscious awareness is categorized, according to a stepwise developmental process beginning with basic awareness of the external environment, followed by awareness of one's body, and finally, higher-order internal awareness of oneself (such as mind-wandering or daydreaming, associated with the Default Mode Network) (97). The emergence of consciousness occurs along a continuum (27) and has been likened to a dimmer switch beginning with a minimum basic consciousness, mediated by subcortical structures (98, 99) and possibly the subplate (4), to higher order consciousness associated with cortical processing and decision-making (Figure 9) (98, 100). Neuroscientists hold that basic conscious awareness requires the subjective ability to evaluate the environment and form coordinated responses (101) and may be demonstrated *via* action planning, learning, and purposeful movement (100).

Fetal neurobehavioral studies analyze observable fetal movements *via* four-dimensional ultrasound or other diagnostic modalities to assess fetal neurologic development (102, 103). Such studies indicate directed actions, motor planning, and learning prior to cortical development. Evidence includes (1) goal-oriented hand movements by 13 weeks gestation (94); (2) differential velocities of fetal hand movements toward the sensitive eye and mouth regions by 22 weeks gestation (104); (3) in twin gestations, evidence of socially-aware motor planning of fetal hand movements toward the co-twin by 14 weeks gestation (105). These studies indicate early action planning, learning, and the emergence of a basic minimum level of consciousness in the fetus by 13–14 weeks gestation. The minimum conscious level marks the starting point of a consciousness that is unreflective, focused on the present, and without a requirement for memory or self-reflection. With further brain development, complex levels of consciousness emerge (106).

The following sections discuss the evidence for and against the hypotheses of cortical necessity and subplate modulation. Tables 2, 3 summarize this evidence.

**Table 2:** A summary of evidence for and against the hypothesis of cortical necessity.

Evidence For	Evidence Against
Neuroanatomical: thalamocortical connections emerge after 24–28 weeks gestation	Pain and consciousness are acknowledged in neonates <24–28 weeks gestation, prior to thalamocortical connections The same indicators of pain that are observable after thalamocortical connections are already present, developing, and maturing prior to this time
Functional: noxious-evoked neural activity is present in the cortical plate after 28 weeks gestation	Brain networks vary by age Neural activity in the subplate, not the cortical plate, is present prior to 24–28 weeks gestation
Adult-like cortical resting state networks are detectable beginning at 30 weeks gestation after thalamocortical connections are established	Brain networks in early human development may not be comparable to adult networks Resting state activity at earlier gestations (<28 weeks) is centered in the subplate
Facial expressions to noxious and non-noxious stimuli are indistinguishable until after 28 weeks gestation when assessing 3 facial responses.	Validated pain scales require assessment of 7–9 facial expressions or facial expressions in combination with physiologic and contextual factors to discriminate pain from non-pain states, particularly in early gestation
Limb withdrawal reflexes may occur to both noxious and non-noxious stimulation prior to 35 weeks gestation	No validated pain assessment tools utilize limb withdrawal as a univariate measure of pain, particularly in early gestations when large receptive fields and lack of descending inhibitory pathways result in lower pain thresholds and increased excitability
Case studies of adult post-lobotomy patients dating from the 1950s, some of whom experienced indifference to pain	Unclear how to correlate variably controlled case studies in the era before neuroimaging to the infant and fetus
	No alteration of pain perception occurs with stimulation or ablation of the somatosensory cortex
	Preserved pain perception in several clinical cases, despite extensive lesions of the cortex on neuroimaging.
	In hydranencephalic children (functionally decorticate), 96% of parents state their child can feel pain

**Table 3:** A summary of evidence for and against the subplate modulation hypothesis.

Evidence For	Evidence Against
Neuroanatomical: subplate circuitry forms the predominant network prior to 24–28 weeks gestation	Lack of primary studies of noxious-evoked subplate activity, due to ethical and technical considerations
Functional: Before 30–32 weeks gestation, electrical activity in the brain is centered in the subplate	
The subplate forms part of the transitional nociceptive circuitry during fetal life	
Neurobehavioral studies indicate subplate modulation of sensorimotor functions begins at 9–10 weeks gestation	
Research demonstrates subplate responsiveness to somatosensory stimuli prior to thalamocortical connections	
Fetal and preterm neonatal responses to noxious stimuli are present prior to thalamocortical connections suggesting the pre-existence of pathways of pain perception	

## The hypothesis of cortical necessity

According to the hypothesis of cortical necessity, there is no capacity to experience pain prior to 24–28 weeks gestation when connections from the thalamus reach the cortical plate (1, 2, 11). Evidence proposed for cortical necessity (Table 2) includes (1) neuroanatomic structural evidence of thalamocortical connections emerging at 24–28 weeks gestation (1); (2) functional evidence of noxious-evoked brain activity in the cortical plate, after 24–28 weeks gestation (1); (3) the presence of resting state networks (spontaneous neural activity) involving the cortex after 28 weeks gestation (2); (4) a reported lack of discriminative facial responses between noxious and non-noxious stimuli before 33 weeks gestation (2); (5) a reported lack of distinction between innocuous touch and noxious-evoked withdrawal reflexes before 35 weeks gestation (2); and (6) case studies of adult post-lobotomy patients dating from the 1950s, some of whom experienced indifference to pain (1, 107).

First, thalamic connectivity to the somatosensory cortex at 24–28 weeks gestation is widely acknowledged. Proponents of cortical necessity state that the cortex is the sole structure that can interpret stimuli as painful; therefore, prior to its development, pain experience is impossible (1, 11). This circular argumentation has been challenged for overreliance on neuroanatomical hypotheses, as was done in the era of untreated neonatal pain, rather than correlation with clinical behavior and other pain indicators which occur before 24–28 weeks gestation. Other researchers note that the same indicators of pain that are present after thalamocortical connectivity are already present, developing, and maturing prior to 24 weeks gestation. No observable behavioral or physiologic indicators at 24–28 weeks gestation have been identified which demonstrate the impact from these connections, suggesting that pre-existing neural pathways mediate pain perception prior to 24–28 weeks.

**Figure 10:**
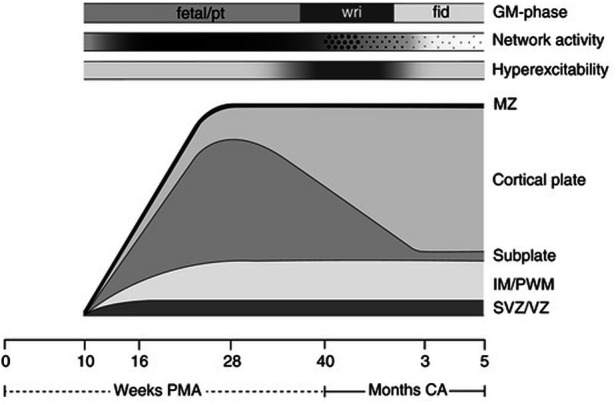
Schematic presentation of the processes underlying the subplate and cortical plate modulation hypothesis (5). The bottom line denotes age, first in weeks PMA, after term (40 weeks) in months (corrected age). Above the age line the developmental changes in the human cortex are depicted. SVZ/VZ represents the subventricular and ventricular zones where the neurons and glial cells are generated; IM/PWM denotes the intermediate zone that gradually develops into the periventricular white matter; MZ is the marginal zone. The following three timelines represent from bottom to top: the hyperexcitability of the nervous system, in which the intensity of the grey shading represents the degree of hyperexcitability; the cortical network activity that emerges across the brain from 9 to 10 weeks PMA, this gradually increases (indicated by increasing shading) to be full-blown present (in the subplate) at mid-fetal age, before moving from global and widespread activity to local and limited activity to local and limited activity (“sparsification”, indicated by the diminution of the dots); on top the developmental changes in general movements. GM, general movements; PMA, postmenstrual age; CA, corrected age; pt, preterm.

Second, noxious stimuli evoke cortical brain activation patterns, or a neurological pain signature (NPS), after 28 weeks gestation. The neurological pain signature, after thalamocortical connectivity, involves the primary and secondary somatosensory, the prefrontal cortex, the anterior cingulate cortex, the amygdala, and the insula. Such cortical activation is present in adults and term infants (29), but is generally absent in extremely preterm infants <28 weeks gestation (91, 108). This is cited as evidence of cortical necessity for pain perception. However, such studies involve testing for a cortical network that does not yet exist in the preterm neonate or fetus <24–28 weeks gestation (Figure 10). Earlier developing subplate circuitry, rather than cortical circuitry, is the predominant network during this time period (22).

Third, resting state networks (RSNs), measurable by functional MRI, are defined as a set of brain regions that show functional connectivity during task-free spontaneous brain activity (109). In 2010, Doria et al. analyzed fMRI of preterm infants to determine when adult-like RSNs could first be detected. At 30 weeks postmenstrual age, fragments of adult-like cortical networks are detectable, increasing to an adult-like repertoire at term age (110). Adult-like RSNs thus emerge during the time period when thalamocortical connectivity is established, subplate circuitry regresses, and cortical circuitry begins to predominate (Figure 10). In 2018, van den Heuvel et al. analyzed fetal resting-state MRI in third trimester fetuses (29–37 weeks gestation), likewise identifying regions or hubs of cortical involvement after thalamocortical connectivity (111). Notably, these studies (1) analyzed fetuses and neonates after 28 weeks, during the phase of cortical plate dominance (Figure 10); and (2) utilized adult resting state networks as the basis of comparison. Brain networks vary by age and may not be comparable to adult networks (Figure 10). At earlier gestations (<28 weeks), fetal resting state fMRI studies indicate that the center of gravity of most activations is located in the subplate zone (13).

Fourth, some researchers suggest that preterm infants and fetuses are incapable of experiencing pain until after 28–33 weeks gestation as they may display nondiscriminative pain-related facial expressions to both noxious and innocuous stimuli at earlier gestations (2). This conclusion is based on a 2019 study of preterm infants, 28–42 weeks postmenstrual age, which utilized a modified PIPP-R pain scale to evaluate 3 facial responses to a control heel lance (which did not pierce the skin) compared to a noxious heel lance used to obtain blood (28). Facial responses were analyzed using a partial PIPP-R pain scale on a 9-point scoring system, compared to the 21-point scoring system in the validated PIPP-R (77). Overall, 24% of infants displayed facial expressions to the non-noxious heel lance, while 69% exhibited facial expressions to the noxious heel lance. Preterm infants <33 weeks gestation were more likely to exhibit pain-related facial expressions to both the control heel lance and the noxious heel lance than those after 33 weeks gestation. Additionally, in 42 of the preterm neonates, EEG responses to both the noxious and non-noxious heel lance were measured, demonstrating (1) nondiscriminative delta brush activity at earlier gestations (<33 weeks), often in response to both noxious and non-noxious heel lance; and (2) increasingly discriminative sensory-evoked potentials and noxious-specific brain activity at later gestations (>34 weeks). Based on this study, some investigators suggest that a sense of pain distinct from benign tactile stimulation does not develop until 32–33 weeks gestation (2).

This conclusion is controversial for the following reasons: (1) the use of 3 facial expressions alone is insufficient to assess pain in early gestations. Rather, per the American Academy of Pediatrics, validated pain scales require assessment of 7–9 facial expressions or multidimensional scoring of behavioral, physiological, and contextual factors to discriminate pain from non-pain states, particularly in extreme prematurity. Increased sensitivity to noxious and non-noxious stimulation at early gestations may be due to sensitization from prior pain experiences (i.e., repeated heel lances), lack of descending inhibitory pathways, and large receptive fields, resulting in lower pain thresholds and increased excitability (6). (2) A subsequent study in 2022 of neonates 28–40 weeks gestation concluded that the use of multimodal pain assessment tools (facial expression, brain activity, heart rate, and limb withdrawal) discriminates noxious from non-noxious procedures in early preterm infants with an accuracy of 78%–79% at 28–31 weeks (61); (3) noxious-evoked brain activity, whether delta brushes or noxious-specific cortical activity, is widely regarded as an unreliable univariate measure of pain, but may hold promise as part of a multimodal pain assessment strategy to discriminate pain from non-pain states (20, 31, 61, 91); (4) It is noteworthy that a heel lancet device was used to test both noxious and non-noxious stimulation. During the non-noxious heel lance, infants experienced not only the tactile stimulation of the lancet device against the heel but also the audible click associated with blade release away from the skin. This raises the question as to whether this method may have triggered anticipatory reactions, particularly in infants previously exposed and perhaps sensitized to noxious heel lances.

Fifth, some researchers report a lack of distinction between noxious and non-noxious evoked limb withdrawal in early preterm infants <35 weeks gestation. In a study by Cornelissen et al., a heel lancet device was also used to deliver both noxious and non-noxious stimuli (see description above), while limb withdrawal responses of the biceps femoris were measured *via* EMG (79). 100% of the infants (30–42 weeks gestation) demonstrated robust withdrawal to the noxious heel lance. However, 29% of the preterm neonates exhibited limb withdrawal in response to the non-noxious heel lance that was indistinguishable in magnitude from noxious-evoked withdrawal. 40% of term neonates likewise responded with limb withdrawal to non-noxious stimulation, but with a significantly smaller magnitude on EMG. The study concludes that flexion reflexes, which may be interpreted as signs of pain in adults, may not reflect pain perception before 35 weeks gestation. Some researchers cite this as evidence that pain perception is unlikely until at least the third trimester (2), however this conclusion is likewise disputed for the use of limb withdrawal as a univariate measure of pain. As previously discussed, multimodal pain assessment tools are necessary to differentiate pain from non-pain states, particularly at early gestational ages.

Finally, case studies of adult post-lobotomy patients dating from the 1950s, some of whom experienced indifference to pain (107), have been cited by the Society for Maternal Fetal Medicine as evidence of cortical necessity for pain perception (1). However, it is unclear how to correlate variably controlled case studies of some adult post-lobotomy patients, in the era before neuroimaging, to the unique structures and mechanisms used for pain processing in fetal and neonatal life (112).

Researchers observe that the primary purpose of acute pain perception is a behavioral drive to survive. This drive serves as a protective mechanism which seeks to remove an individual from a damaging stimulus and is critical for the survival of the organism. As such, scientists expect neural circuitry responsible for this drive to be in phylogenetically older regions of the brain, which appear earlier in development (113). Such regions include the subplate and subcortical structures such as the thalamus and brainstem. Cortical regions, which are phylogenetically newer, may be involved in the processing and regulation of pain, rather than pain perception itself (114, 115).

Evidence for the role of cortical regions in the processing and modulation of pain, rather than pain perception *per se* includes (1) the presence of validated pain indicators prior to connections to the cortex (6); (2) several clinical cases of preserved pain perception despite lesions of critical regions including the insula, anterior cingulate, and even the entire contralateral hemisphere, confirmed by neuroimaging (114–116); (3) no alteration of pain perception with stimulation or ablation of the somatosensory cortex, while altered pain perception occurs with stimulation or ablation of the thalamus (117); (4) cases of infants and children with hydranencephaly (congenitally decorticate) who demonstrate pain-related responses as well as elements of consciousness (118–120); notably, 96% of parents of children with hydranencephaly stated that their child can feel pain (119); (5) research in term infants showing the top-down inhibitory effects of the prefrontal cortex, anterior cingulate cortex, and the anterior insula, as part of the DPMS, in dampening the pain experience and modifying pain behavior (29). This indicates that noxious-evoked cortical activity may represent modulation and regulation of pain rather than perception of pain itself.

The hypothesis of cortical necessity has also been challenged for (1) disregarding clinical practice in neonatology and anesthesiology in which pain is acknowledged and treated in neonates prior to thalamocortical connectivity (6); and (2) relying on neuroanatomical studies from post-mortem fetuses without functional correlation to fetal behavior *in utero* (93, 121).

The presence of pain in the preterm infant prior to thalamocortical connectivity has been widely acknowledged for decades and challenges the assumption of cortical necessity (Table 2). Per the American Academy of Pediatrics, prevention and management of pain in the earliest preterm infants is the standard of care, not only to prevent short- and long-term adverse consequences but to alleviate pain itself (6). In one survey of NICU clinicians, all agreed that preterm babies born at the edge of viability (21–23 weeks) are able to perceive pain and demonstrate the same signs of pain that older patients demonstrate when they are in pain (122). Neonatal pain researchers acknowledge the urgent need for improved pain assessment and management in the youngest premature infants to prevent underdiagnosis and undertreatment of pain (19, 27, 29–31). This suggests that (1) the hypothesis of cortical necessity is not congruent with clinical practice; and (2) pain perception may be mediated by functional and well-developed pathways prior to thalamocortical connections.

## The subplate modulation hypothesis

The cortical subplate forms part of the transitional nociceptive circuitry during fetal life and is present in all placental animals to varying degrees. The subplate is conspicuously present in primates, particularly in humans, during early neurological development (5). Initially, the subplate was thought to be the structural analog of a warehouse, filled with neurons awaiting migration to the cortical plate (73). More recently, evidence suggests that subplate circuitry is more akin to a power station than a warehouse, forming a functionally responsive network of early cortical activity in the first through third trimesters (5, 73, 123, 124). The subplate emerges at 8 weeks gestation, reaching maximum thickness around 28 weeks (124). When thalamic fibers reach the cortical plate at 24 weeks, the gradual transition between subplate circuitry and cortical circuitry begins, reaching completion in the post-term period (5, 125). This results in two overlapping developmental phases (Figure 10) characterized by:

(a) the transient cortical subplate phase, ending at 3 months post-term when the permanent circuitries in the primary motor, somatosensory and visual cortices have replaced the subplate; and subsequently,
(b) the phase in which the permanent circuitries dominate [(22), p. 276].

This has been called a transitional pain circuitry of the fetus and neonate, similar to the transitional fetal and neonatal circulatory system (126). Before 30–32 weeks, electrical activity in the brain is centered around the subplate and is marked by the presence of delta brushes and spontaneous activity transients on EEG (28, 127, 128).

Evidence supporting the subplate as an active and functional precursor of the cortex includes modulatory activity of both sensorimotor and somatosensory functions prior to thalamic innervation of the cortical plate: (1) neurobehavioral studies indicate subplate modulation of fetal motor activity beginning at 9–10 weeks gestation (129); (2) research demonstrates subplate activation in response to sensory stimuli (130); (3) pain indicators prior to 24 weeks gestation suggest the pre-existence of pathways of pain perception (Table 3).

First, fetal neurobehavioral research indicates subplate modulation of sensorimotor activity during early gestation. Subplate modulation in the fetal and preterm periods followed by cortical plate modulation in the post-term period has been observed in the study of general movements (GMs), the most common motor behavior of the fetus and neonate (5, 129). Research indicates that subplate modulation of GMs predominates from 9 to 34 weeks gestation until the immature but progressively developing cortical plate circuitry takes over in the post-term period. The dissolution of the subplate at 3 months post-term marks the completed transition to permanent cortical plate circuitry. In the post-term period, when cortical activity in the primary sensorimotor cortex shifts from subplate to cortical plate, observable changes in motor behavior occur with the emergence of so-called fidgety movements (Figure 10). This evidence indicates that the sensorimotor region of the subplate forms an active and functioning cortical network beginning as early as 9–10 weeks gestation.

Second, in the somatosensory region of the subplate, animal research in ferrets indicates (1) subplate neurons are the first cortical neurons to respond to auditory stimuli; (2) the subplate shows topographic organization, comparable to the cortical plate; that is to say, sensory stimulation of a particular area evokes changes in predictable anatomic location in the subplate (130). Preterm human infants, likewise, show early responsiveness to external stimulation with light flashes, demonstrating evoked delta brush responses associated with subplate circuitry (131). This indicates that both the sensorimotor and somatosensory regions of the subplate are responsive and active during this developmental phase. Functional magnetic resonance imaging of the fetal subplate *in utero* corroborates these findings. At 20 weeks gestation (the earliest gestation studied), the brain region with the highest activity is the cortical subplate (5, 13).

Finally, fetal and preterm neonatal responses to noxious stimuli predate thalamocortical connections at 24 weeks gestation (Figure 1). The presence of an early pain circuitry is highly suggested by the occurrence of the same pain-related responses before and after 24 weeks gestation. These indicators include facial expressions of pain, vigorous body movements, and physiologic and hormonal stress responses that are mitigated by analgesics. The subplate forms the most significant functional network during the preterm age, is functionally active in the sensorimotor and somatosensory regions of the subplate (5, 130), and has been implicated in early responses to painful stimulation (14).

The predominant criticism of the subplate modulation hypothesis is the limited direct evidence that the subplate and subcortical structures modulate pain perception in addition to modulating other sensorimotor or somatosensory functions (12). It is accurate that noxious-induced testing of the human subplate during corticogenesis is limited by ethical, technical, and legal considerations (132). If the subplate is an active and functional precursor of the cortex corresponding in topography, then subplate and subcortical modulation of other somatosensory functions, such as pain perception, may be anticipated. Additional research opportunities may be possible during therapeutically-indicated noxious procedures in extremely preterm infants and during intrauterine fetal surgery.

## Discussion

While the acknowledgment of pain perception in the fetus prior to thalamocortical connectivity at 24–28 WGA is controversial, the acknowledgment of pain in the age-matched preterm infant is not. In clinical practice, the extremely preterm infant is the focus of extensive research efforts to better assess and treat pain in the neonatal intensive care unit. Numerous studies acknowledge that pain in the preterm infant is underrecognized and undertreated with focused research on ways to better ameliorate pain in this population (19, 27, 29–31).

Research and clinical practice indicate that fetal pain perception is possible prior to thalamocortical connectivity *via* pre-existing pathways of pain perception. Some researchers argue that neither consciousness nor pain capacity exists prior to 24–28 weeks gestation (1, 2, 11). Advances in the fields of neonatology, fetal surgery, fetal anesthesiology, and fetal neurobehavior make this viewpoint no longer appropriate. However, a fetal pain paradox continues to exist in which pain-related responses in the extremely preterm infant are regarded as evidence of pain, while the same responses to noxious stimuli in a similarly-aged fetus are dismissed as reflexive responses, not indicative of a pain experience.

Determining the exact onset of pain perception in the fetus is challenging. Fetal responses to therapeutically indicated noxious procedures are evident by 15–16 weeks gestation and are alleviated by analgesics. Prior to this time frame, published research is lacking. Researchers acknowledge that “where it is uncertain whether harm may result, it is advisable to apply a precautionary principle that errs on the side of caution to prevent potential harms, even if scientific uncertainty exists about their extent [(28), p. 498].” Certainly, such a viewpoint is prudent to avoid the errors of the past.

With the exponential increase in invasive fetal procedures, particularly in North America, it is surprising that more studies of fetal responses to noxious stimuli are not available. This is a field worthy of further investigation, as we will not see what we do not look for. A systematic review of 165 fetal surgical studies with over 5,000 fetal surgical procedures observed that none of these studies fully analyzed fetal reactions to tissue-damaging procedures *via* intraoperative fetal monitoring, such as fetal movements, fetal hormonal responses, and heart rate variability (81). A lack of standardized dosage regimens of direct fetal anesthesia was also noted, with dosages of opioids varying by hundreds of micrograms per kilogram, raising the question of inadequate analgesia. This is noteworthy, as there is increasing evidence that painful procedures early in life are instrumentally harmful in altering pain sensitivity and cognition later in life (28).

Implications of fetal pain perception at earlier gestational ages include the need for research in fetal pain assessment and management strategies to ensure adequate procedural and post-procedural pain control (3, 57, 58) and to prevent adverse short- and long-term sequelae, including the potential for preterm labor (1, 18). An ethical obligation also exists to prevent, mitigate, and treat pain whenever it can be anticipated. Finally, informed consent regarding fetal pain capacity is an important ethical consideration. The informed consent process should distinguish between the surgical procedures, anesthesia, and analgesia utilized for the pregnant woman and those utilized for the fetus. Reports over the past 20 years indicate that the potential for pain perception in the fetus is a concern for women and families (34, 122, 133), which has implications for fetal surgery as well as abortion.
